# A Study of Adhesion in Foamed WMA Binder-Aggregate Systems Using Boiling Water Stripping Tests

**DOI:** 10.3390/ma14216248

**Published:** 2021-10-20

**Authors:** Anna Chomicz-Kowalska

**Affiliations:** Department of Transportation Engineering, Faculty of Civil Engineering and Architecture, Kielce University of Technology, Al. Tysiąclecia Państwa Polskiego 7, 25-314 Kielce, Poland; akowalska@tu.kielce.pl

**Keywords:** adhesion, foamed bitumen, warm-mix asphalt, boiling water stripping test, digital image analysis, FTIR, dynamic viscosity

## Abstract

The paper investigates the phenomena of adhesion in binder-aggregate systems produced to reflect warm-mix asphalt with water-foamed bitumen. The investigated materials included limestone and quartzite aggregates and a total of four asphalt binders: 50/70 and 45/80–55 bituminous binders obtained from two sources. The adhesive bonding between the asphalt binders and aggregates was evaluated in boiling water stripping tests, which results were quantified using digital image analysis. The bituminous binders were additionally tested for their dynamic viscosities, and their chemical composition was probed using FTIR spectroscopy. The tests were carried out using traditional liquid and foamed bituminous binders on samples prepared at temperatures characteristic of hot-mix asphalt and warm-mix asphalt production (20 °C decrease). The use of foamed binders yielded higher values of residual asphalt binder coverage of the aggregates. Limestone aggregates provided superior adhesion, with the lowest result amounting to approximately 88%, while with quartzite aggregates, the results ranged from approximately 40% to 87%. The refinery from which the asphalt binders were sourced had a significant influence on the results; however, the additional rheological and chemical analyses were insufficient to explain those differences. It was concluded that the process of asphalt binder foaming, per se, may have a beneficial impact on the resistance of the asphalt binder-aggregate system to the action of water.

## 1. Introduction

In recent years, new materials and techniques have been introduced in the road construction industry, permitting a shift toward more energy-efficient and sustainable transport infrastructure. Incentives toward these goals are provided by government strategies toward emission reductions such as ‘The Roadmap to a Resource Efficient Europe’ put forward by the European Commission (EU). These new developments include the introduction of reclaimed materials and industrial by-products [1,2,3,4,5,6,7,8,9], reinforcement [10,11,12,13], use of decreased processing temperatures [14,15,16], and novel approaches to mix design [17,18]. The warm-mix technologies were first introduced in the 1990s, and over the years, they have proven to mostly perform on par with comparable hot mixes [14,19,20]. Nevertheless, some concerns regarding the early service performance of these mixtures remain [21].

Warm-mix technologies allow for significant reductions in emissions and energy consumption associated with road construction [14]. Typically, mixtures recognized as warm-mix asphalt (WMA) are produced at temperatures 20 to 30 °C lower than equivalent hot mixtures. Different methods are used to obtain the required workability and compactability of those mixtures: asphalt binder additives [16,22], asphalt binder foaming [23,24], asphalt mix additives [25,26,27,28], binder fluxing [29], and combinations of the mentioned measures [23,30,31,32].

The use of mechanical water foaming permits the production of warm-mix asphalts without any other additives, while other techniques usually have some built-in antistripping measures. This fact provides a possibility for investigating the moisture resistance of such mixtures without the confounding effects of antistripping agents. It should be noted, though, that antistripping agents are usually used in water-foamed mixtures as an obligatory measure for increasing the pavement durability, just as in hot-mix asphalt. Many studies have been recently published regarding the effects of foaming on the properties of bituminous binders. The majority of the works focus on the functional properties of the foamed binders, mostly showing no significant detrimental effects of the foaming process; however, decreased aging of the binders is often raised as a potential problem for the short-term high-temperature performance of these mixtures [33,34,35,36,37,38,39,40,41,42]. A number of studies have been published on the resistance to moisture damage of warm-mix-asphalt produced using mechanical water foaming, showing that these mixtures may be more susceptible to the action of water [43,44,45,46]. In some studies [44], it was also shown that plant-produced foamed warm-mix asphalt exhibits similar moisture susceptibility to hot-mix asphalt, as opposed to laboratory-produced warm mixes. It was also demonstrated that the amount of aging may have significant effects on the moisture resistance of warm-mix asphalts, proving that adequate laboratory aging [47] or summer aging before the winter period [48] may be sufficient to improve this characteristic.

Although the moisture susceptibility tests involving compacted asphalt mix specimens tend to represent the performance of the final mixture quite well, their results may be confounded by additional factors such as degree of compaction, mixture gradation, mixture composition, or even the conditioning protocol (e.g., MIST vs. freeze-thaw as reported in the work of [44]). Such factors are excluded in model studies, such as the binder-aggregate affinity tests using different methods [49]. These methods, in general, can be separated into three groups: rolling bottle methods (typically carried out at room temperature), static methods (at room or elevated temperatures), and boiling water stripping methods. All mentioned tests exhibit disadvantages, e.g., strong mechanical action in the rolling bottle method, long testing time, and weak differentiation of static tests, while the boiling water methods are troublesome and may be inaccurate due to the use of calibration curves and chemical reagents [50]. A number of authors have used digital image analysis for assessing the results of these tests with great accuracy and confidence [51,52,53,54,55,56,57].

Based on the presented literature in the subject area, a study was conducted to investigate the effects of mechanical water foaming on the stripping performance of different aggregate-bitumen systems in model studies, using the boiling water stripping method and digital image analysis method for evaluating the results. The boiling water stripping method was chosen as being among the least influenced by the mechanical properties of the aggregate (in opposition to the rolling bottle method) and due to its short duration, which was important given the extensive testing program. Additionally, asphalt binder tests were conducted to explore the possible sources of the variance in the results of the stripping tests. Tests for evaluating dynamic viscosity were conducted in relation to its role in the surface energy theory of aggregate-bitumen adhesion and its mechanical resistance to flow during the boiling tests, while the FTIR analyses were also conducted to evaluate the presence of carboxylic and sulfoxide compounds contributing to the moisture susceptibility of the binders [58,59,60].

## 2. Materials and Methods

### 2.1. Design of Experiment

The study involved boiling water stripping tests and basic asphalt binder tests to evaluate the impact of bitumen foaming and the decreased mixing temperatures on the aggregate-bitumen bonding. A full factorial 2^5^ experimental design with three replicates (three boiling tests) was conducted. The study totaled 32 different experiments and required 96 boiling water stripping tests to be performed. The factors included in the design were as follows:

- Type of the asphalt binder:
  ○ PGB—50/70 paving-grade bitumen
  ○ PMB—45/80–55 polymer-modified bitumen
- Source of the asphalt binder:
  ○ A—source A
  ○ B—source B
- Form of the binder while it was added to the aggregates:
  ○ N—non-foamed
  ○ F—foamed
- Temperature at which the aggregates were coated:
  ○ H—hot, corresponding to hot-mix asphalt mixing temperatures:
    ▪ 150 °C when the 50/70 PGB binders were used
    ▪ 165 °C when the 45/80–55 PMB binder were used
  ○ W—warm, corresponding to warm-mix asphalt mixing temperatures:
    ▪ 130 °C when the 50/70 PGB binder were used
    ▪ 145 °C when the 45/80–55 PMB binder were used
- Type of the aggregate used:
  ○ L—limestone
  ○ Q—quartzite

The experimental plan has been shown in the form of a matrix in Table 1.

The asphalt binders were selected based on their broad use in asphalt mixtures for surface courses subjected to all kinds of traffic. The asphalt binders were subjected to dynamic viscosity testing and chemical analysis using infrared spectroscopy.

The mixing temperatures corresponding to the production technology of hot-mix asphalt were established based on EN 12697-35 [61] standard in case of the paving-grade bitumen and based on the manufacturer’s recommendations in the case of the polymer-modified binders. The temperatures corresponding to warm-mix asphalt technology were set 20 °C lower than the established hot-mix temperatures. Asphalt binder-aggregate adhesion was measured indirectly using boiling water stripping tests and evaluated by digital image analysis.

### 2.2. Materials

The investigation used a total of four asphalt binders used widely in surface courses: 50/70 penetration paving-grade bitumen and 45/80–55 polymer (elastomer) modified bitumen (as classified in accordance with the EN 12591 [62] and EN 14023 [63], respectively), sourced commercially from two different local refineries Lotos Asfalt (Gdańsk, Poland) and Orlen Asfalt (Płock, Poland). The basic characterization of asphalt binders in accordance with appropriate standards is given in Table 2.

The aggregates used in the study were sourced commercially from limestone and quartzite quarries: Sitkówka-Trzuskawica S.A (Nowiny, Poland) and Wiśniówka-Eurovia (Zagnańsk, Poland), respectively. The characterization of aggregates is given in Table 3.

### 2.3. Methods

#### 2.3.1. Sample Preparation and Execution of Boiling Water Stripping Tests

The testing protocol used in this study was based on the method formalized in the EN 12697-11, Section 7: boiling water stripping method [68].

The first step of the sample preparation was to conduct foaming of the asphalt binders. The bitumen was foamed using WLB10S laboratory foamer (Wirtgen, Windhagen, Germany) using 2% foaming water content as in the work of [37], transferred to glass containers, and cooled to ambient temperature. Non-foamed binders were also poured into such containers and cooled.

**Figure 1:**
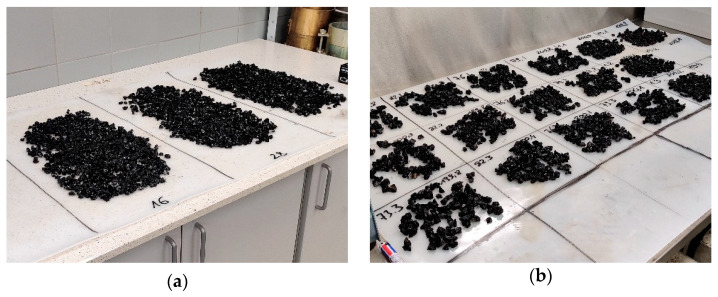
Sample preparation process: (**a**) coated aggregates left for cooling; (**b**) samples after the boiling water stripping test.

The aggregates were sieved to obtain 8/11 mm size samples, rinsed with water, dried to constant mass at 105 °C, and bagged in 1500 g portions. To coat the aggregates with asphalt binders, they were both heated to the mixing temperature increased by 5 °C, and subsequently mixed in pre-heated steel bowls using glass rods. The bitumen was added to the aggregates in a 2% ratio by mass, corrected for the aggregate density by a factor of α = 2650/ρ_p_, where ρ_p_ is the aggregate density in Mg/m^3^. The aggregates were mixed thoroughly with the binder so that all the particles were completely coated by the asphalt binder, which was always achieved in less than 3 min of mixing. The mixture was then laid on a silicone rubber mat, the coated particles were separated using the glass rod and left for cooling at ambient temperature (Figure 1a).

Three 200 g ± 0.5 g samples were prepared for boiling tests from each of the coated aggregate batches. The boiling water stripping tests were conducted in line with EN 12697-11. After the boiling tests, samples were transferred onto silicone rubber mats. The aggregate particles were separated and left for cooling and drying for a time period of a minimum of 24 h (Figure 1b).

#### 2.3.2. Digital Image Capture and Analysis

Based on previous trials and the experiences of other researchers [51,52,53,54,55,56,57], a methodology for acquiring high-quality photographic data was developed, main principles of which included:

- Image acquisition under repeatable, controlled, artificial lighting using a ring light;
- Use of a distinct background color for ease of its removal in post-processing, should it be necessary;
- Placing the sample aggregates without gaps for mitigating the background reflections;
- Each sample was photographed once, then it was flipped, and its underside was photographed again to obtain more data per sample; this also would decrease any potential bias of the initial sample arrangement.

**Figure 2:**
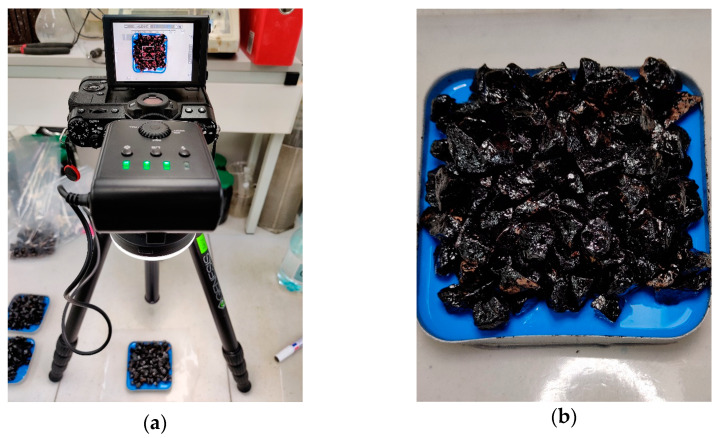
Acquisition of digital images: (**a**) setup used for the digital imaging; (**b**) sample prepared for digital imaging.

The capture of digital images of the aggregate samples was carried out using a consumer photographic mirrorless camera with an APS-C CMOS III sensor (Fujifilm, Tokio, Japan). The camera was placed on a tripod, looking straight down on the photographed sample (Figure 2a). The optics were set at a focal length of 55 mm and f/11 aperture to mitigate distortions and provide adequate depth of field. Directly to the lens, two accessories were attached: a circular polarizing filter (for controlling reflections on the bitumen surface) and a ring light (primary light source). The exposure settings were set to omit clipping of highlights and shadows, and a reference target was used to equalize all exposures in post-processing.

The aggregate samples, which were previously subjected to the boiling water stripping tests, were carefully arranged on small trays filled with silicone rubber in a total of 2 to 3 layers, ensuring that the background (silicone rubber) would not reflect off the asphalt binder coating the aggregates (Figure 2b). The samples were photographed once, and after they were flipped, the arrangement of the particles was corrected so that a second photograph could be taken. The samples were packaged and archived.

**Figure 3:**
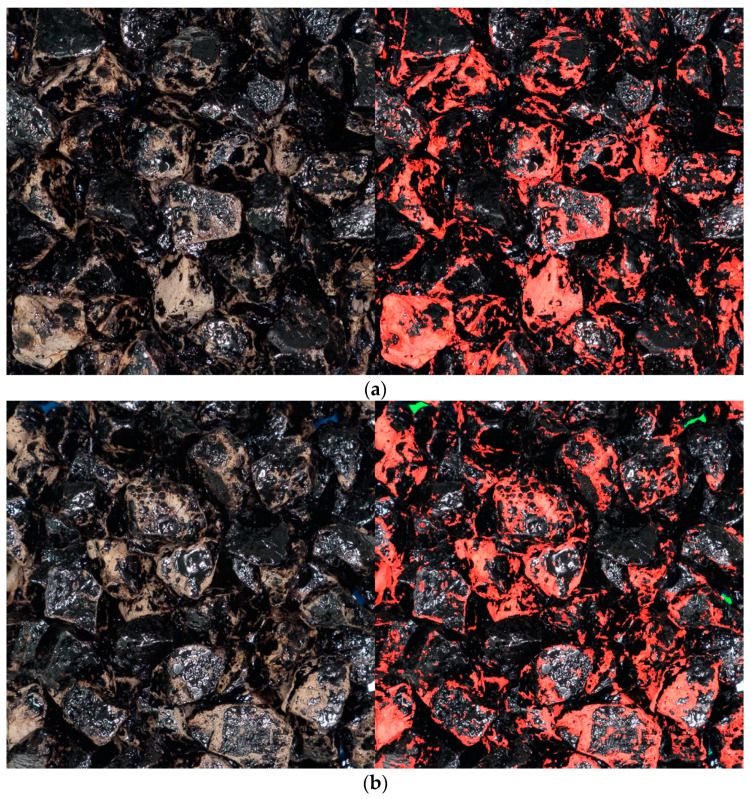
Exemplary results of PMB-B-N-H-Q experiment image analysis: (**a**) first photograph—78.7% coverage; (**b**) second photograph after sample flipping and rearranging of aggregate particles—79.3% coverage; final sample coverage—79.0%.

Before the actual analysis was performed, the images were subjected to preprocessing to equalize the exposure characteristics and cropped to view only the central part of the sample. The images were analyzed using a Python [69] script and the OpenCV package [70]. The original RGB (red, green, blue) images were analyzed using the HSV (hue, saturation, value) color model used widely in computer vision and image analysis [71,72]. Using this method, the image areas corresponding to the background, aggregates covered with asphalt binder, and to stripped aggregate surface were identified and verified using color masks (Figure 3). Based on these masks, the pixel counts were obtained for those regions. The areas calculated from two pictures, taken before and after sample flipping (Figure 3a,b), were added, and asphalt binder coverage of the visible aggregate surface was calculated.

#### 2.3.3. Conventional Testing of Bituminous Binders

**Figure 4:**
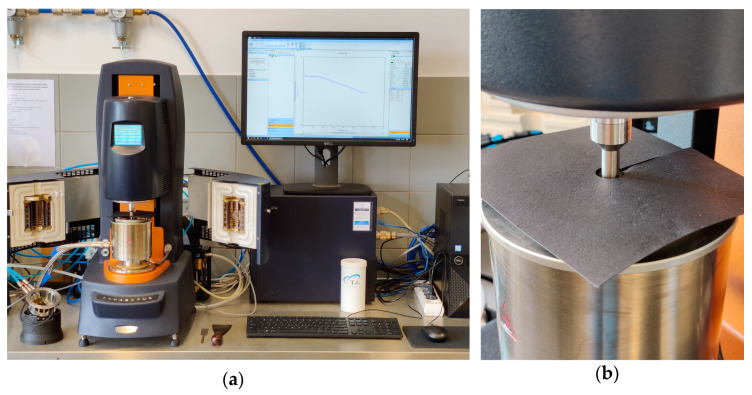
TA Instruments DHR-2 rheometer: (**a**) the device; (**b**) a protecting cover in case of spontaneous bursts of foamed bitumen.

Dynamic viscosity (EN 13302) was measured using a DHR-2 dynamic shear rheometer (Discovery Hybrid Rheometer, 2019, TA Instruments, New Castle, DE, USA) in rotating spindle (coaxial cylinders) configuration as shown in Figure 4a, with three replicates. While testing the foamed binders, the DSR had to be protected from spontaneous bursts of the foamed bitumen, which were found to be occurring at temperatures exceeding 130 °C due to a very small testing geometry gap (Figure 4b). This phenomenon also inhibited the testing of foamed binders at temperatures in excess of 145 °C. The dynamic viscosity measurements were conducted at a 1/s shear rate from the highest to the lowest temperature.

#### 2.3.4. Fourier-Transform Infrared Spectrometric Analysis

In this study, attenuated total reflectance Fourier-transform infrared (ATR-FTIR) spectroscopy was used to evaluate the composition of the foamed binders. The Thermo-Scientific Nicolet iS 5 FTIR Spectrometer (Waltham, MA, USA) and the PIKE Technologies GladiATR (Madison, WI, USA) attenuated total reflectance accessory with a diamond window was used. The bitumen samples after foaming were poured into 1 dm^3^ glass containers, reheated at 140 °C for 2 h, and homogenized for uniform distribution of water during the first stages of cooling at room temperature. The testing was conducted 3 days after foaming. Samples were transferred onto the ATR crystal mechanically, without reheating. Three different samples of each binder were tested, and the potential effects of foaming on the binders’ chemical composition, specifically due to oxidative aging, were measured by evaluating the formation of sulfoxide and carbonyl compounds [73]. Additionally, the presence of elastomeric additives was investigated in their specific absorption bands.

The quantitative evaluation of the mentioned compounds was conducted by calculating normalized indices as given in Table 4. The areas under the respective peaks were computed by the common tangent baseline method as proposed in the work of [74].

## 3. Results

The results are shown in the form of bar plots, with the bars representing the means of the measured values and the width of whiskers (‘error bars’) corresponding to the width of 95% confidence intervals. The values printed on the figures also refer to the mean measured values and 95% CI’s.

### 3.1. Properties of the Asphalt Binders

**Figure 5:**
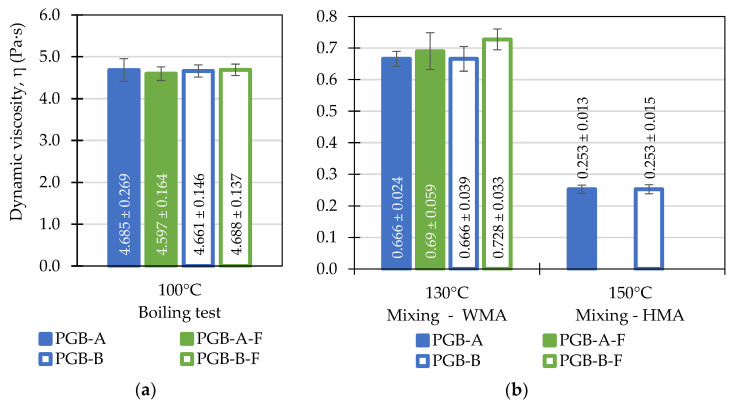
Results of the dynamic viscosity testing of the investigated paving-grade bitumen (PGB) before and after foaming (F) evaluated at: (**a**) 100 °C—the temperature of the boiling water stripping tests; (**b**) 130 and 150 °C—the aggregate-binder mixing temperatures.

**Figure 6:**
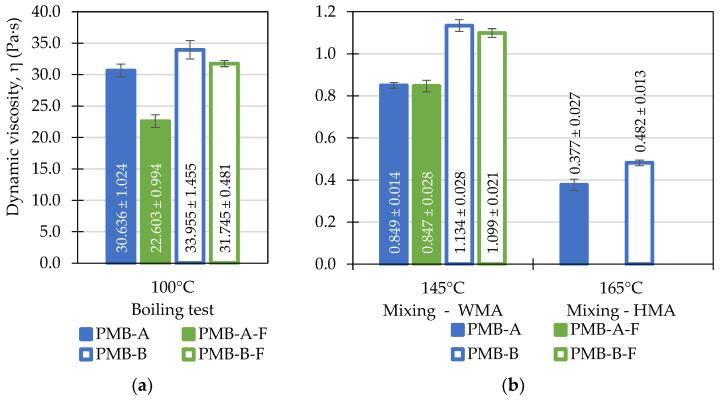
Results of the dynamic viscosity testing of the investigated polymer-modified bitumen (PMB) before and after foaming (F) evaluated at: (**a**) 100 °C—the temperature of the boiling water stripping tests; (**b**) 145 °C and 165 °C—the aggregate-binder mixing temperatures.

The results of dynamic viscosity tests are presented in Figure 5 and Figure 6. The presented values were measured at different temperatures corresponding to the temperatures used in the preparation and execution of the boiling water stripping tests: 100 °C corresponding to the boiling tests, 130 and 150 °C as when mixing of the paving-grade bitumen with aggregates, 145 and 165 °C as when mixing of the polymer-modified bitumen with aggregates. In principle, higher values of dynamic viscosity at the temperature of the boiling test and lower values at the mixing temperature should improve the stripping performance.

As it can be seen in Figure 5, the paving-grade bitumen binders obtained from two different suppliers presented similar viscosity characteristics in the investigated temperature range. As shown in Table 2, the binders were also very similar in terms of penetration and softening point. The non-foamed binders exhibited nearly identical values of dynamic viscosity. It was also observed that foaming had little influence on this property of both asphalt binders. The mean values of dynamic viscosity of the foamed binders were slightly higher than in the case of the non-foamed bitumen, but the variance in the data was too large (specifically at 130 °C) to permit any strict conclusions in this regard. It was observed after the testing that both foamed paving-grade binders presented little foaming potential.

Figure 6 presents the results of dynamic viscosity testing of the polymer-modified bitumen binders sourced from the two refineries. In this case, the differences in dynamic viscosities of these binders were more pronounced, with the PMB-B binder exhibiting higher η values. What is more, the process of foaming had different effects in this case. Both polymer-modified binders exhibited significant drops in dynamic viscosity after foaming at the temperature of the boiling stripping test (100 °C). At higher testing temperatures, this effect was less visible.

**Figure 7:**
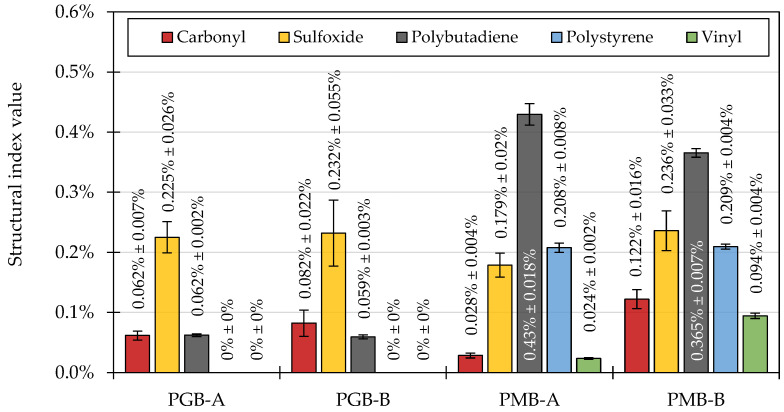
Values of the structural indices calculated based on the ATR-FTIR spectra of the investigated binders.

Figure 7 presents the values of the structural indices based on the measurements of ATR-FTIR spectra of the investigated paving-grade and polymer-modified binders sourced from both refineries.

**Figure 8:**
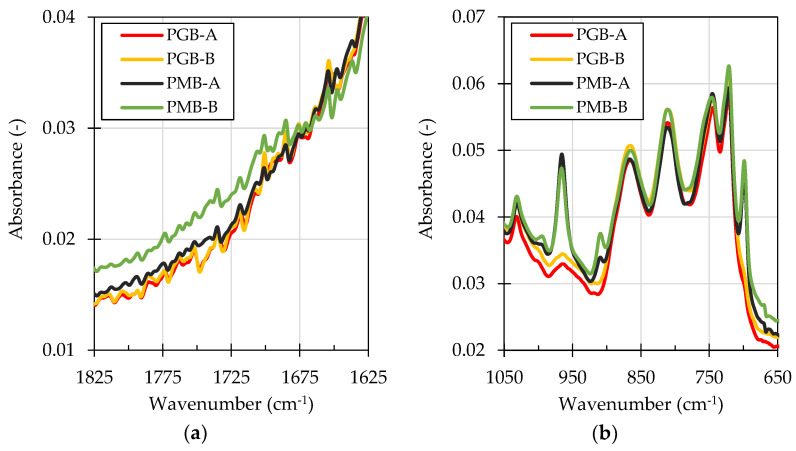
Infrared (ATR-FTIR) absorbance spectra of the evaluated binders in the 1625–1825 cm^−1^ (**a**) and the 650–1050 cm^−1^ (**b**) wavenumber ranges.

The two paving-grade bitumen binders showed similar chemical characteristics, which correlates with their performance in penetration, softening point, and dynamic viscosity tests. Both PGB binders showed similar values of the carbonyl and sulfoxide indices. The peaks in the carbonyl region were mostly found in the 1660–1715 cm^−1^ region (Figure 8a), characteristic of ketone compounds [73,81,82].

The PMB-A and PMB-B binders were more different in their chemical characterization. Higher concentrations of carbonyl and sulfoxide compounds were found to be present in the PMB-B binder. The ratios of the calculated polybutadiene, polystyrene, and vinyl indices also were different in the two PMBs, probably as a result of different polymers being used for their modification (Figure 8b).

### 3.2. Results of the Boiling Water Stripping Tests

**Figure 9:**
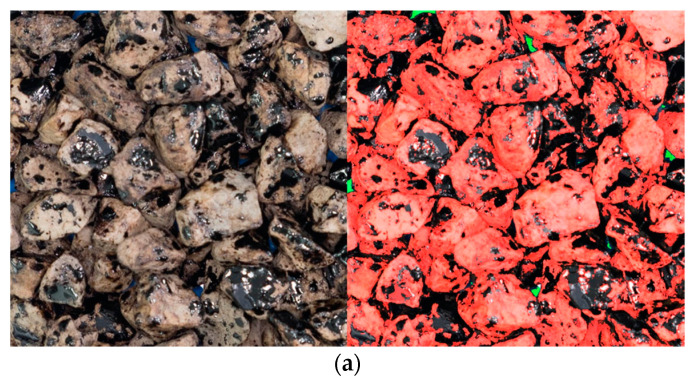

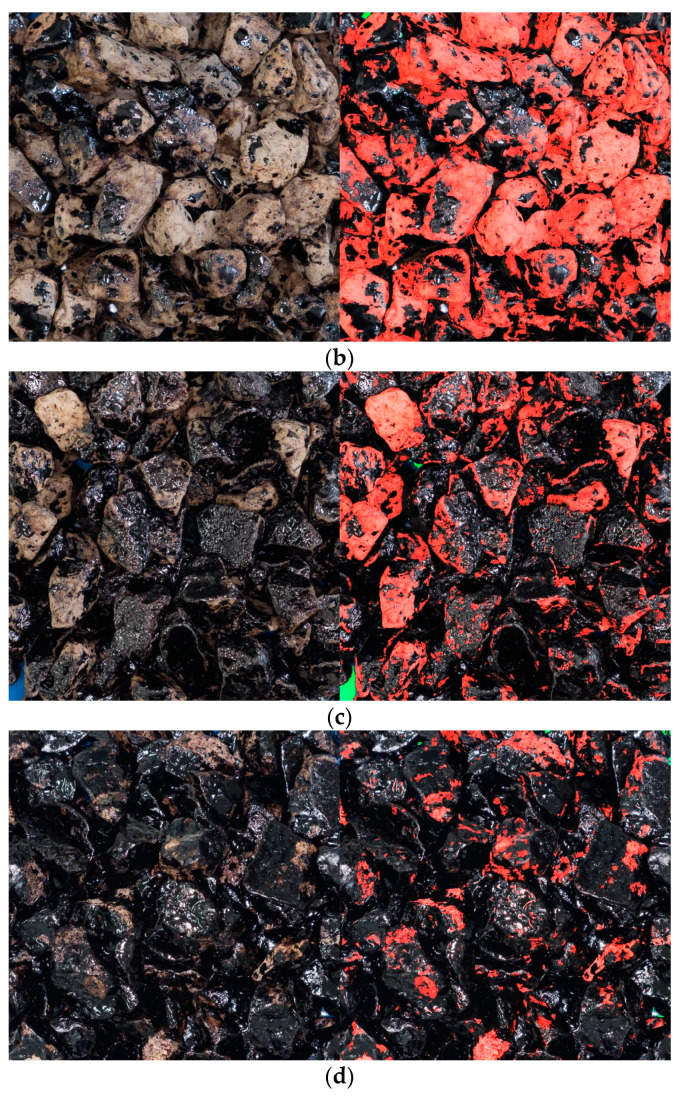
Exemplary results of the image analysis: (**a**) PGB-A-N-W-Q sample—34% coverage; (**b**) PGB-A-F-W-Q sample—47.6% coverage; (**c**) PMB-A-N-H-Q sample—79.9% coverage; (**d**) PGB-B-F-W-L sample—90.8% coverage.

Figure 9 presents exemplary results of the image analysis conducted on binder-coated aggregate samples after the boiling water stripping tests. The figure presents different samples of limestone and quartzite aggregates characterized by a range of approximately 34% to 91% binder coverage, in increasing order.

**Figure 10:**
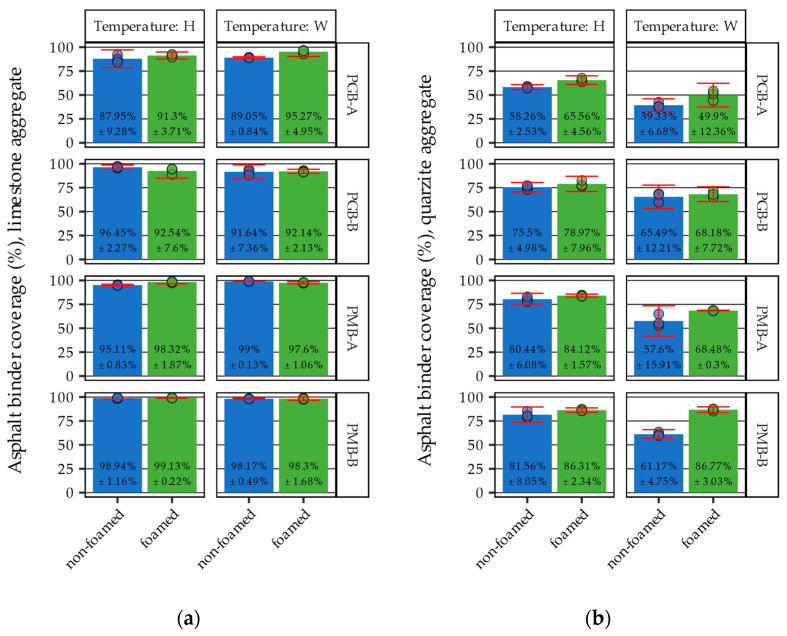
Results of boiling water stripping tests: (**a**) limestone aggregate; (**b**) quartzite aggregate.

The results of the conducted boiling water stripping tests are presented in Figure 10 in the form of boxplots with individual test values shown as dots and 95% confidence intervals (whiskers).

The residual asphalt binder coverage was significantly affected by all the evaluated factors: type of the binder, source of the binder, form of the binder, temperature of mixing, and above all, the type of the aggregate. The variability of the results was significantly influenced by the aggregate. When limestone aggregate was used, the values of residual asphalt binder coverage ranged from 87.95% ± 3.73% to 99.00% ± 0.20%, whereas in the case of quartzite aggregate, these values ranged from 39.33% ± 6.68% to 86.77% ± 3.03%. This shows that the limestone aggregate enabled very strong bonding with the bitumen binders in all of the investigated cases. On the other hand, the aggregate-bitumen systems with quartzite aggregate were significantly more susceptible to the changing parameters of the coating process. When quartzite aggregates were used, the effects of binder type, its source, its form, and coating temperature could be clearly observed. The asphalt binders from source ‘A’ returned lower values of residual asphalt binder coverage. In addition, lowering the mixing temperatures resulted in a decrease in the coverage values. It was found that this effect could be partially mitigated by the use of the binder in the form of foam, as the experiments with foamed binders yielded higher values of residual asphalt binder coverage compared to the typical liquid binder. It can also be concluded that polymer-modified binders provided better resistance to the displacing action of water.

When evaluating the effects of asphalt binder foaming and the mixing temperatures, in many cases, it can be seen that the experiments involving foamed asphalt binder and WMA temperatures resulted in residual asphalt binder coverage comparable to those obtained in experiments representing typical hot mixtures (non-foamed binder, higher mixing temperatures). The use of water foaming in many of those instances (usually when asphalt binder from source B was used) compensated for the decreased coating temperatures.

## 4. Discussion

The conducted boiling water stripping tests permitted the evaluation of the investigated factors on the asphalt binder-aggregate bonding, specifically in the case of quartzite aggregates. Clear differences could be observed in the performance of the asphalt binders based on their source. Both types of the binders obtained from source B, the paving-grade bitumen and polymer-modified bitumen, provided stronger adhesion to the aggregates. This was most strongly visible in the case of the quartzite aggregate, but this statement is also true for limestone aggregates. The results of the rheological tests and chemical analysis provided little explanation for this outcome.

The biggest differences in the residual asphalt binder coverage were seen in the PGB binders, which differed the least in the values of dynamic viscosity across all evaluated temperatures. On the other hand, the stripping performance of the polymer-modified binders was more similar; however, their dynamic viscosities differed greatly.

The results of the chemical analysis were also inconclusive in terms of discriminating the performance of the binders from the two sources. According to some authors [58,59], the carbonyl-band compounds and sulfoxides present in the bitumen are among the most strongly adsorbed on the aggregate surfaces. It is most often reported that carboxylic acids and sulfoxides are most easily displaced by water, while ketones have the least propensity to be displaced by water among the strongly adsorbed compounds. Although these functional groups were identified in the evaluated asphalt binders, their concentrations were low, typical of neat binders [37]. Additionally, the carbonyl indices of the evaluated binders were mainly shaped by the absorption bands of ketones (Figure 8a). In other studies, it was also reported [83] that even asphalt binders known to contain ketones and sulfoxides (RTFO-PAV aged) may perform well in terms of resistance to water action. It is also known that among the most significant factors influencing the moisture resistance of asphalt binders in binder-aggregate systems, other than the aggregate type, is the nitrogen content in the binder [58] and use of caustics during asphalt binder production [58,66], which were not evaluated in this study.

## 5. Conclusions

The present paper provided novel findings regarding the impacts of water foaming on the phenomena of asphalt binder-aggregate adhesion in a model study including boiling water stripping tests. The study evaluated additional factors, including the type of the binder, its source, mixing temperature, and type of the aggregate based on its affinity to bituminous binders.

The presented results showed that the mechanical water foaming of the bitumen improved its resistance to the displacing action of water, regardless of the source, type, and chemical characteristics of the binder. These observations were confirmed in limestone and quartzite aggregates, with hot-mix and warm-mix mixing temperatures used. The experiments have also shown that the decreased mixing temperature had visibly detrimental effects on the adhesive bond between the aggregates and asphalt binder, specifically in the case of quartzite aggregates.

The tests have also shown that the performance of the investigated bituminous binders was highly dependent on their source (producer), despite vary similar basic, chemical (based on FTIR analysis), and dynamic viscosity characteristics.

The findings of this study may put in new perspective several prior experimental works regarding the moisture susceptibility of warm-mix asphalts produced with foamed bitumen.

A number of technical observations were made based on the present research, previous trials, and the experiences of other researchers [51,52,53,54,55,56,57]:

- Repeatable lighting conditions with uniform lighting and mitigation of shadows are recommended;
- Careful selection and preparation of the workplace is required to mitigate color contaminated light reflections (e.g., blue color from the sky, other colors from clothes, furniture, etc.);
- Precise arrangement of the aggregate particles without visible gaps is highly recommended for reliable classification of the reflective surfaces of asphalt binder; when gaps are present, the background reflects off the asphalt binder, which makes it difficult to distinguish from the binder, regardless of the background color;
- When artificial lighting is used and color analysis is made, use of lighting with high color reproduction index (CRI) is advised.

## Figures and Tables

**Table 1:** Characterization of the paving-grade and polymer-modified bitumen used in the study.

No.	Asphalt Binder Type	Asphalt Binder Source	Form of the Asphalt Binder	Mixing Temperature	Aggregate Type
1	PGB	A	non-foamed	hot	limestone
2	PGB	A	non-foamed	hot	quartzite
3	PGB	A	non-foamed	warm	limestone
4	PGB	A	non-foamed	warm	quartzite
5	PGB	A	foamed	hot	limestone
6	PGB	A	foamed	hot	quartzite
7	PGB	A	foamed	warm	limestone
8	PGB	A	foamed	warm	quartzite
9	PGB	B	non-foamed	hot	limestone
10	PGB	B	non-foamed	hot	quartzite
11	PGB	B	non-foamed	warm	limestone
12	PGB	B	non-foamed	warm	quartzite
13	PGB	B	foamed	hot	limestone
14	PGB	B	foamed	hot	quartzite
15	PGB	B	foamed	warm	limestone
16	PGB	B	foamed	warm	quartzite
17	PMB	A	non-foamed	hot	limestone
18	PMB	A	non-foamed	hot	quartzite
19	PMB	A	non-foamed	warm	limestone
20	PMB	A	non-foamed	warm	quartzite
21	PMB	A	foamed	hot	limestone
22	PMB	A	foamed	hot	quartzite
23	PMB	A	foamed	warm	limestone
24	PMB	A	foamed	warm	quartzite
25	PMB	B	non-foamed	hot	limestone
26	PMB	B	non-foamed	hot	quartzite
27	PMB	B	non-foamed	warm	limestone
28	PMB	B	non-foamed	warm	quartzite
29	PMB	B	foamed	hot	limestone
30	PMB	B	foamed	hot	quartzite
31	PMB	B	foamed	warm	limestone
32	PMB	B	foamed	warm	quartzite

**Table 2:** Characterization of the paving-grade and polymer-modified bitumen used in the study.

Binder Type	PGB-A (50/70)	PGB-B (50/70)	PMB-A (45/80–55)	PMB-B (45/80–55)
Penetration, EN 1426 [64] (0.1 mm)	56.6	55.8	56.5	56.7
Softening Point, EN 1427 [65] (°C)	49.4	49.5	63.3	66.2
Frass Breaking Point, EN 12593 [66] (°C)	−13	−15	−19	−19

**Table 3:** Characterization of the limestone and quartzite aggregates used in the study in accordance with EN 13043 [67].

Aggregate Type	Limestone	Quartzite
Apparent Particle Density, ρ_a_ (Mg/m^3^)	2.71	2.65
Oven-Dried Particle Density, ρ_rd_ (Mg/m^3^)	2.65	2.58
Saturated and Surface-Dried Particle Density, ρ_ssd_ (Mg/m^3^)	2.69	2.60
Water Absorbtion, WA (%)	0.57	0.4
Resistance to Fragmentation, LA	23	21
Resistance to Polishing for Application in Surface Courses, PSV	38	58
Resistance to Wear, M_DE_	13	7
Fines Content, f (%)	1.1	1.8

**Table 4:** Structural indices calculated for the bituminous binders [75,76,77,78,79,80].

Structural Index	Bond	Characteristic Peak Wave Number (cm^−1^)	Chemical Index Expression:
Sulfoxide	S=O, stretching	1030	$I_{S=O}=\frac{A_{1030}}{\sum A_{all}}$
Carbonyl	C=O, stretching	1700	$I_{C=O}=\frac{A_{1700}}{\sum A_{all}}$
Polybutadiene	C-H, oop bending of trans-alkene	966	$I_{PB}=\frac{A_{966}}{\sum A_{all}}$
Polystyrene	C-H, oop bending in monoakrylated aromatic	699	$I_{PS}=\frac{A_{699}}{\sum A_{all}}$
Vinyl	=C-H oop bending in vinyl groups	990, 910	$I_{Vi}=\frac{A_{910}+A_{990}}{\sum A_{all}}$

Σ*A_all_ = A_(2953, 2923, 2862)_ + A_1700_ + A_1600_ + A_1460_ + A_1376_ + A_1310_+ A_1030_ + A_990_ + A_966_ +A_910_ + A_864_ + A_814_ + A_743_ + A_724_ + A_699_*. oop—out of plane.

## Data Availability

Data available on request.
